# Insights on decomposition process of c-C_4_F_8_ and c-C_4_F_8_/N_2_ mixture as substitutes for SF_6_

**DOI:** 10.1098/rsos.181104

**Published:** 2018-10-17

**Authors:** Ying Zhang, Yi Li, Xiaoxing Zhang, Song Xiao, Ju Tang

**Affiliations:** 1 Scientific Research Institute of Electric Power, Guizhou Power Grid Company Ltd, Guiyang 550002, People's Republic of China; 2 School of Electrical Engineering, Wuhan University, Wuhan 430072, People's Republic of China

**Keywords:** c-C_4_F_8_/N_2_, ReaxFF-MD, density functional theory, decomposition

## Abstract

In recent years, many scholars have carried out studies on c-C_4_F_8_ and its gas mixture and found it has potential to be used as an environment-friendly insulating medium to replace SF_6_ in medium-voltage equipment. In this paper, the c-C_4_F_8_ and c-C_4_F_8_/N_2_ gas mixture models were built to study its decomposition process by the combination of reactive molecular dynamics method and density functional theory. The yield of the main decomposition products, the reaction pathways and enthalpy under different temperatures were explored. It was found that the decomposition of c-C_4_F_8_/N_2_ mainly produces CF_2_, F, CF_3_, CF, C, CF_4_ and C_2_F_4_. c-C_4_F_8_ can decompose to C_2_F_4_ by absorbing 43.28 kcal/mol, which is the main decomposition path and this process easily occurs under high temperature. There is a dynamic equilibrium process among the various produced radicals, which ensures the insulation performance of system to a certain extent. The decomposition performance of c-C_4_F_8_/N_2_ mixture is better than that of pure c-C_4_F_8_ at the same temperature. Relevant results provide guidance for engineering application of the c-C_4_F_8_/N_2_ gas mixture.

## 1. Introduction

Nowadays, electrical equipment using SF_6_ as the insulation medium occupies a dominant position in the field of medium-voltage (MV) and high-voltage (HV) application. About 80% of the SF_6_ gas produced worldwide is used in HV circuit breakers (GCB) and in gas-insulated switchgear [1]. However, the atmospheric lifetime of SF_6_ is up to 3200 years and its global warming potential (GWP) is 23 500 times than that of CO_2_. Over the past 5 years, the global atmospheric content of SF_6_ has increased by 20% and its atmospheric mole fraction reaches to 7.28 ppq (part(s) per quadrillion) currently corresponding to a radiative forcing of 0.0041 W m^−2^ [2,3]. In addition, SF_4_, SO_2_F_2_, SO_2_, SOF_2_ and other products produced by the decomposition of SF_6_ under long-term operating conditions are toxic substances, which pose a threat to equipment maintenance personnel [4]. With the increasing demand in environmental protection around the world, the carbon emission of power industry has also been strictly limited. Therefore, it is urgent to seek for an environmentally friendly gas as insulation medium for power industry.

At present, scholars have made some achievements on environmentally friendly insulation medium such as perfluorocarbons (PFCs), trifluoroiodomethane (CF_3_I), fluoroketones (FKs), fluoronitriles and their gas mixture [5–8]. Among them, CF_3_I is a moderately toxic gas and can precipitate iodine element after discharges. Particulate iodine may cause corrosion to the equipment to a certain extent, which limits the application of CF_3_I [9,10]. FKs have a crude formula of the form C*_n_*F_2*n*_O. C_5_F_10_O and C_6_F_10_O are the two main FKs with the liquefaction temperatures of 26.9°C and 49°C under normal pressure and thus need to be used with other gases with lower liquefaction temperature [11]. Fluoronitriles contain CN group in their molecular structure and may produce toxic substances. PFCs mainly include c-C_4_F_8_, C_3_F_8_, C_2_F_6_ and CF_4_. The insulation performance of c-C_4_F_8_ reaches 1.1 times than that of SF_6_, and its GWP value is 8700 [12]. Many scholars have carried out experimental and theoretical research on c-C_4_F_8_ and its gas mixture. It was found that the insulation performance of c-C_4_F_8_/N_2_, c-C_4_F_8_/CO_2_ and c-C_4_F_8_/CF_4_ gas mixture is great, indicating that c-C_4_F_8_ gas mixture has immense potential for use in MV equipment [12–14].

The internal insulation of the electrical equipment is ageing under normal operating conditions. And it is inevitable to produce a variety of insulation defects, leading to partial discharge (PD) or flashover and decomposition of insulating medium. Thus, the evaluation of the decomposition characteristics of gas-insulated medium is of great significance. Several achievements have been made in the research on the decomposition characteristics of c-C_4_F_8_ under a discharge and local overheating faults. *Li et al*. tested the decomposition products of c-C_4_F_8_/N_2_ gas mixture under PD, spark discharge and arc discharge. They found that CF_4_, C_2_F_6_, C_2_F_4_, C_3_F_8_ and C_3_F_6_ are the main decomposition products [15]. Hayashi *et al.* explored the reaction mechanism of CF_2_ particles produced by c-C_4_F_8_ based on density functional theory (DFT) and revealed the dissociation properties of c-C_4_F_8_ molecules comprehensively [16]. Cobos *et al*. investigated the thermal decomposition characteristics of c-C_4_F_8_ at 1150–2300 K. It is found the decomposition of c-C_4_F_8_ firstly produces two C_2_F_4_ molecules, and C_2_F_4_ can further dissociate producing CF_2_ particles [17].

In this paper, the decomposition mechanism of c-C_4_F_8_ and c-C_4_F_8_/N_2_ gas mixture was investigated by the combination of reactive molecular dynamics method and DFT. We built the c-C_4_F_8_ and c-C_4_F_8_/N_2_ models to explore the decomposition process of c-C_4_F_8_ gas mixture under different temperatures. The yield of the main decomposition products, the reaction pathways and enthalpy under different temperatures were also obtained. Relevant results provide guidance for engineering application of c-C_4_F_8_/N_2_ gas mixture.

## 2. Methods

The development of reactive molecular dynamics method provides an effective way to study the physical and chemical properties of large-scale system (millions of atoms). Reactive force field (ReaxFF) describes bond cleavage and formation based on the bond level, which originates from the distance between two atoms. ReaxFF has been widely used in the field of pyrolysis, combustion and catalysis [18–22]. The terms of total energy in ReaxFF can be described as the following equation [23]:

2.1 $$E_{\mathrm{system}}=E_{\mathrm{bond}}+E_{\mathrm{over}}+E_{\mathrm{under}}+E_{\mathrm{val}}+E_{\mathrm{pen}}+E_{\mathrm{tors}}+E_{\mathrm{conj}}+E_{\mathrm{vdWaals}}+E_{\mathrm{Coulomb}},$$

where *E*_bond_ denotes the bond energy; *E*_over_ and *E*_under_ correspond to the over and under coordinated atom in the energy contribution, respectively; and *E*_val_, *E*_pen_, *E*_tors_, *E*_conj_, *E*_vdwaals_ and *E*_Coulomb_ represent the valence angle term, penalty energy, torsion energy, conjugation effects to energy, non-bonded van der Waals interaction and Coulomb interaction, respectively.

**Figure 1.**
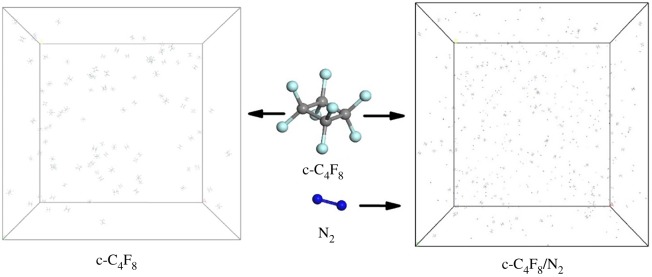
Representative snapshots of c-C_4_F_8_ and c-C_4_F_8_/N_2_ system (light blue for F atom, grey for C atom and dark blue for N atom).

In order to explore the decomposition mechanism of c-C_4_F_8_ and c-C_4_F_8_/N_2_ gas mixture, two periodic cubic models were built (as shown in figure 1). It is reported that the highest allowable pressure of 20%c-C_4_F_8_/80% N_2_ gas mixture at −20°C and −30°C is about 0.35 and 0.2 MPa, respectively [24]. And most MV equipment working at 0.15–0.3 MPa. In order to explore the decomposition mechanism of c-C_4_F_8_/N_2_ mixture at this scale, we built models with 20% c-C_4_F_8_ and 80% N_2_. The width of the c-C_4_F_8_ system is 155 Å, which contains 100 c-C_4_F_8_ molecules with the density about 0.008918 g cm^−3^. The width of c-C_4_F_8_/N_2_ system is 265 Å, which contains 100 c-C_4_F_8_ molecules and 400 N_2_ molecules with the density about 0.00274 g cm^−3^. The above parameters correspond to the actual density of the gas mixture at 0.1 MPa, 25°C.

The system was minimized for 5 ps at 5 K using the NVE (keep the number of atoms, volume and potential energy constant) ensemble and then equilibrated with the NVT (keep the number of atoms, volume and temperature constant) ensemble for 10 ps at 1000 K using a time step of 0.1 fs [22]. Then the NVT (keep the number of atoms, volume and temperature constant) molecule dynamics simulations were performed at different temperatures for 1000 ps with the time step of 0.1 fs. The Berendsen thermostat method with a 0.1 ps damping constant was used to control the temperature [25]. All the ReaxFF-MD simulations were carried out using the Amsterdam density functional package, and the force field file is given in the data availability section [26].

In addition, quantum chemistry DFT calculation was performed to obtain the reaction enthalpy of the main decomposition paths at different temperatures [27]. The geometry optimization of the reactants and products for each path is performed using the double numerical atomic orbital augmented by d-polarization (DNP) as the basis set. The exchange–correlation energy is described using the meta-generalized approximation (mGGA-M06 L) function [28]. Geometry optimizations of all the particles were performed using the convergence threshold of 1.0 × 10^−5^ Ha on energy, 0.005 Å on displacement and 0.002 Ha Å^−1^ on gradients. We also did zero-point energy (ZPE) correction and enthalpy correction based on the frequency analysis to obtain more accurate results. All the DFT calculations in this paper were conducted using DMol^3^ package of the Materials studio.

## 3. Results and discussion

### 3.1. Decomposition rate of c-C_4_F_8_ and c-C_4_F_8_/N_2_ gas mixture

Local overheating, PD and arc discharge are the common failures in electrical equipment [29]. PD and arc discharge are mostly caused by insulation defects in the devices. And the temperature in the central region of the PD and arc discharge is about 1000 K and 3000–12 000 K, respectively [30,31]. High temperature will lead to the decomposition of insulating medium, producing various free radicals or decomposition products. The generation of decomposition products may affect the insulation performance of the gas-insulated medium and cause threat to the equipment. In this paper, we carried out the reactive molecular dynamics simulations of c-C_4_F_8_ and c-C_4_F_8_/N_2_ system at different temperature conditions to explore its decomposition mechanism.

**Figure 2.**
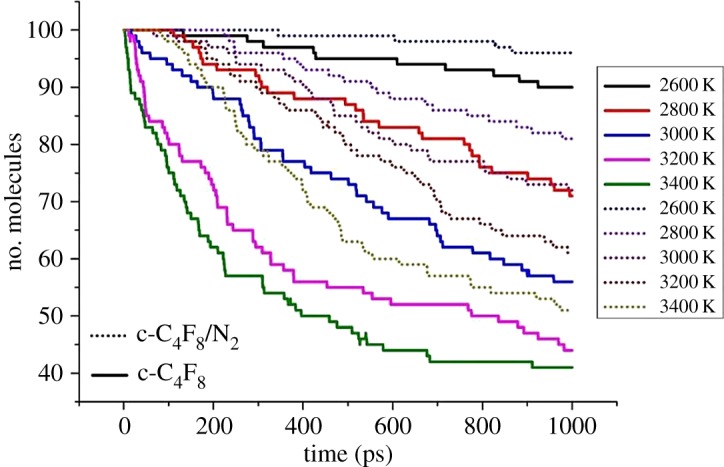
Time evolution of c-C_4_F_8_ decomposition at 2600–3400 K.

**Figure 3.**
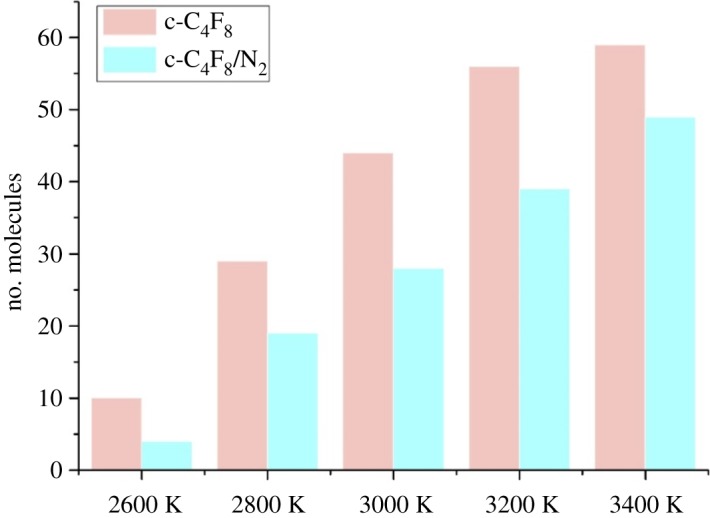
Maximum number of decomposed c-C_4_F_8_ at 2600–3400 K.

Figures 2 and 3 describe time evolution of c-C_4_F_8_ decomposition in pure c-C_4_F_8_ and c-C_4_F_8_/N_2_ systems and the maximum number of decomposed c-C_4_F_8_ at 2600–3400 K, respectively. It should be noted that in order to allow chemical reactions to be observed on the computational affordable time scale, we enhanced the temperatures to accelerate the simulation process. We have tested and found that c-C_4_F_8_ and c-C_4_F_8_/N_2_ mixture begin to decompose largely at 2600 K (figure 2). The decomposition rate of c-C_4_F_8_ shows an increasing trend with the increase of temperature. The decomposition rate of c-C_4_F_8_ in the pure c-C_4_F_8_ system is significantly accelerated above 3000 K. The final decomposition amount and the decomposition rate of c-C_4_F_8_ in c-C_4_F_8_/N_2_ system are lower than that of pure c-C_4_F_8_ system at the same temperature, which indicates that the decomposition characteristics of c-C_4_F_8_/N_2_ gas mixture is great. For example, only 49 c-C_4_F_8_ decomposed in c-C_4_F_8_/N_2_ system at 3400 K, whereas 59 c-C_4_F_8_ molecules decomposed in c-C_4_F_8_ system under the same condition. In addition, the density of c-C_4_F_8_ system (0.008918 g cm^−3^) is higher than that of c-C_4_F_8_/N_2_ system (0.00274 g cm^−3^). Thus the molecules of c-C_4_F_8_ in the unit volume increase, resulting in the increase of the effective collision number and the intensity of reactions. And the decomposition amount of c-C_4_F_8_ in the c-C_4_F_8_ system is higher than that of c-C_4_F_8_/N_2_ system at the same temperature.

**Figure 4.**
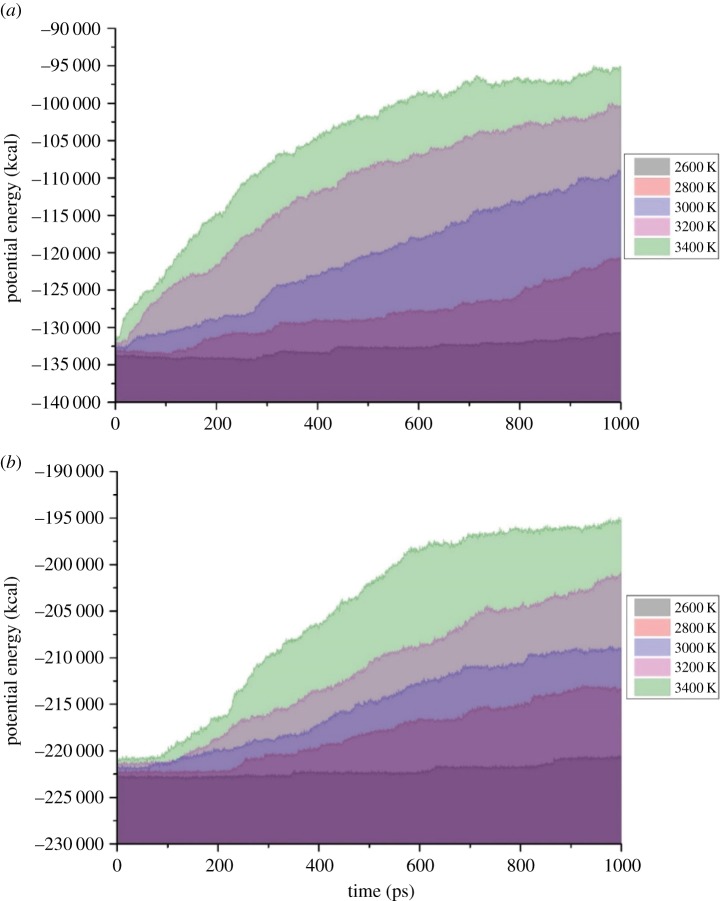
Time evolution of potential energy at 2400–3400 K in c-C_4_F_8_ system and c-C_4_F_8_/N_2_ system.

Figure 4 shows time evolution of potential energy at 2400–3400 K in c-C_4_F_8_ and c-C_4_F_8_/N_2_ system. It can be seen that the potential energy shows an increasing trend in the whole simulation process, indicating that the decomposition process of c-C_4_F_8_ and c-C_4_F_8_/N_2_ gas mixture is endothermic. The total potential energy and its growth rate increases with the increase of temperature. The potential energy of c-C_4_F_8_ system has no obvious change when the ambient temperature is at 2600 K, which is due to the insufficient occurrence of various reactions at this temperature. When the ambient temperature reaches above 3200 K, the potential energy of the system increases rapidly in the time range of 0–400 ps, and exhibits a saturated growth trend after 400 ps. This means the decomposition of c-C_4_F_8_ is concentrated at 0–400 ps. The time evolution of potential energy in c-C_4_F_8_/N_2_ system is basically the same as that of c-C_4_F_8_ system.

On the whole, the decomposition performance of c-C_4_F_8_/N_2_ mixture is better than that of pure c-C_4_F_8_ at the same temperature, which is suitable to use as a gas-insulated medium in the field of MV equipment.

### 3.2. Distribution of decomposition products

**Figure 5.**
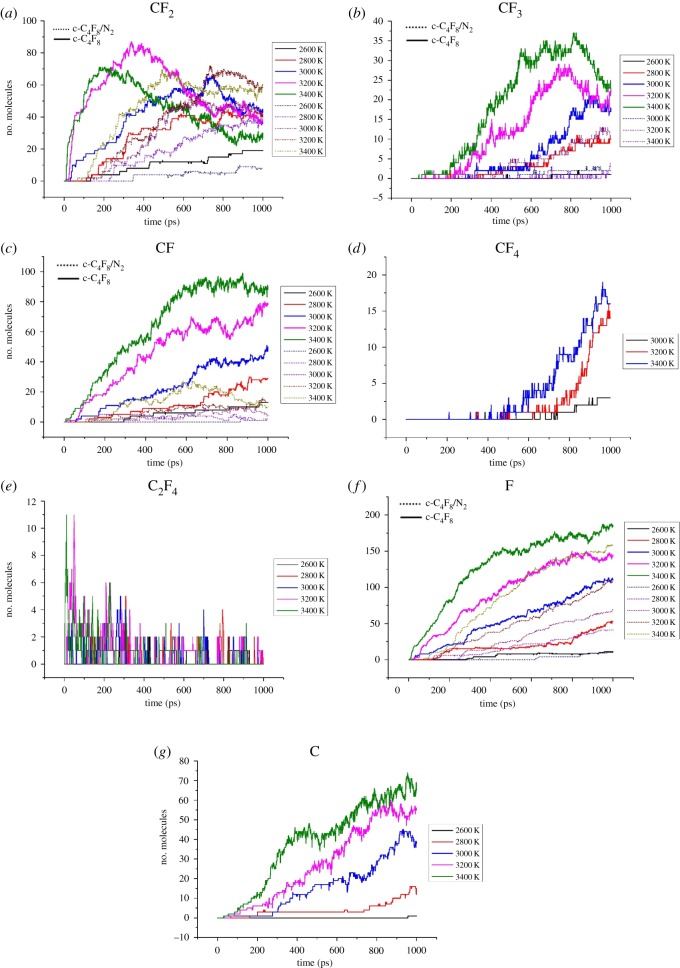
(*a–g*) Time evolution of c-C_4_F_8_ decomposition products at 2600–3400 K.

The distribution of the main decomposition products in the c-C_4_F_8_ and c-C_4_F_8_/N_2_ system is shown in figure 5. It can be found that decomposition of c-C_4_F_8_ mainly produces CF_2_, CF_3_, CF, F, C, C_2_F_4_ and CF_4_.

For the c-C_4_F_8_ system, the yields of CF, F and C show a linear increase trend with the increase of temperature. The two groups of free radicals, CF_2_ and CF_3_ show a saturated growth trend at temperatures below 3200 K. When the temperature is higher than 3200 K, the yield of CF_2_ decreased in the range of 400–1000 ps and the yield of CF_3_ decreased in the range of 600–1000 ps, which is relative to the re-decomposition of CF_2_ and CF_3_ particles at high temperature. The yield of CF_4_ increased significantly at temperatures above 3200 K. The generation of CF_4_ requires the participation of CF_3_, thus the decrease of CF_3_ content is related to the formation of CF_4_. The yield of C_2_F_4_ reached its peak at the beginning of the simulation at 3200 and 3400 K, and then began to decrease. In addition, C atoms are also found during the simulation. It should be noted that particulate carbon is detrimental to the insulation properties of the system.

The yields of the main decomposition particles in the c-C_4_F_8_/N_2_ system are lower than that of pure c-C_4_F_8_ system at the same temperature. The content of CF_2_ shows a saturated growth trend when the temperature is above 3200 K. The generation of CF_3_ begins at 3000 K and its content is relatively low. The time evolution of CF and F radicals is similar to that of c-C_4_F_8_ system. In addition, the yields of C_2_F_4_ and C are much lower than those of pure c-C_4_F_8_ system at the same temperature.

**Figure 6.**
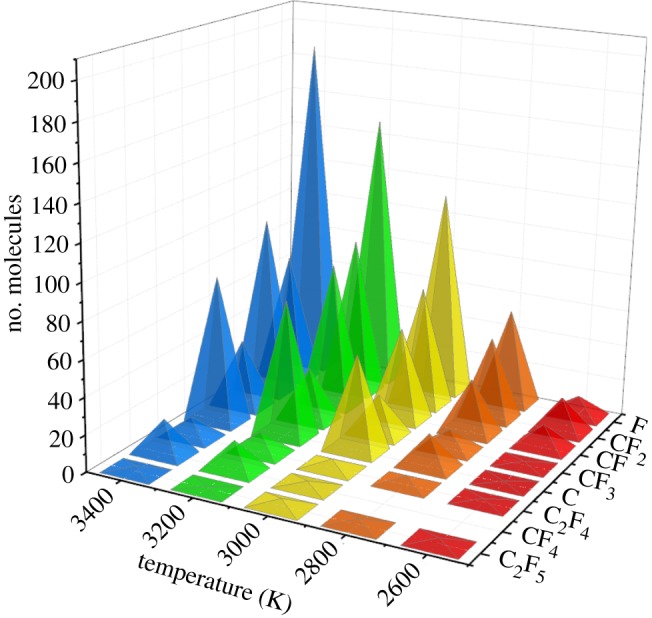
Maximum number of produced decomposition products of c-C_4_F_8_ at 2600–3400 K (c-C_4_F_8_ system).

**Figure 7.**
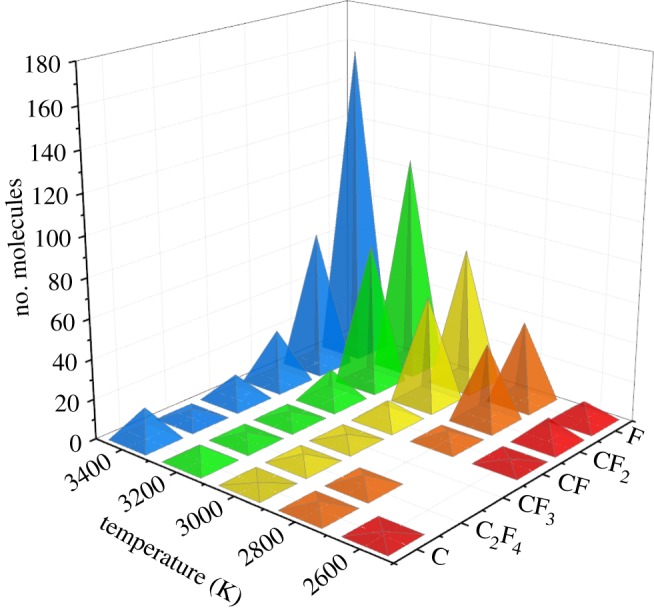
Maximum number of produced decomposition products of c-C_4_F_8_ at 2600–3400 K (c-C_4_F_8_/N_2_ system).

The maximum number of produced decomposition products of c-C_4_F_8_ and c-C_4_F_8_/N_2_ system is shown in figures 6 and 7, respectively. It can be found that the content of F in the C_4_F_8_ system is the highest among all the decomposition products, followed by CF_2_, CF and C. The content of F and CF_2_ is the highest in the c-C_4_F_8_/N_2_ system and the content of CF_3_ is relatively low among all the decomposition products.

### 3.3. Decomposition mechanism of c-C_4_F_8_

**Figure 8.**
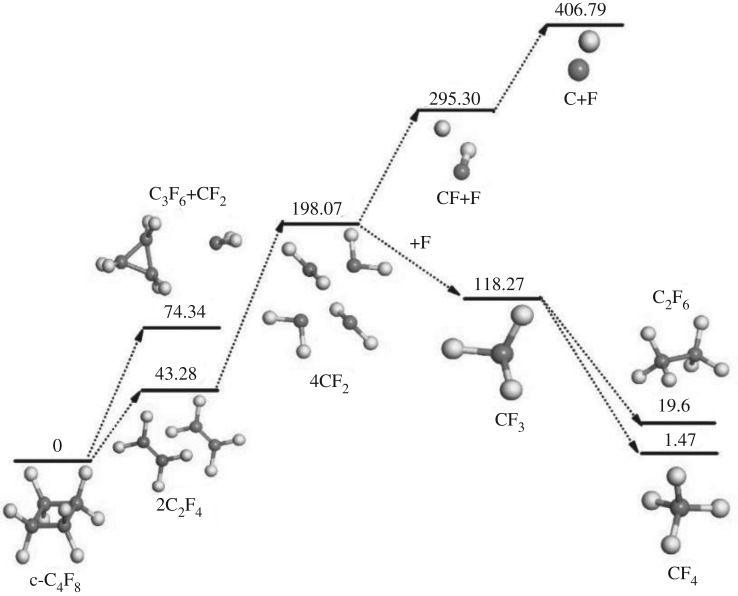
Relative energy change of c-C_4_F_8_ decomposition process.

The proposed decomposition mechanism and reaction enthalpy of c-C_4_F_8_ molecule based on the ReaxFF-MD simulation results are shown in table 1 and the relative energy change of c-C_4_F_8_ decomposition process is shown in figure 8. It can be found that the generation of C_2_F_4_ needs to absorb 43.28 kcal mol^−1^, which is more prone to occur than the formation of C_3_F_6_ and CF_2_. C_2_F_4_ can further decompose to two CF_2_ radicals, which needs to absorb 77.93 kcal mol^−1^. As the main decomposition product of c-C_4_F_8_, CF_2_ can also dissociate to produce CF and F or combine with F to generate CF_3_, and these processes need to absorb 97.23 kcal mol^−1^ or release 79.80 kcal mol^−1^, respectively. In addition, the formation of CF_4_ and C_2_F_6_ releases 116.8 and 98.67 kcal mol^−1^, respectively. And the decomposition of CF requires to absorb 111.49 kcal mol^−1^.

**Table 1.** Proposed decomposition mechanism and reaction enthalpy of c-C_4_F_8_.

no.	reaction	enthalpy (kcal mol^−1^)^a^
1	$c-C_{4}F_{8}\to 4CF_{2}$	198.07
2	$c-C_{4}F_{8}\to 2C_{2}F_{4}$	43.28
3	$c-C_{4}F_{8}\to C_{3}F_{6}+{\mathrm{CF}}_{2}$	74.34
4	$CF_{2}\to \mathrm{CF}+F$	97.23
5	CF→C+F	111.49
6	$CF_{2}+F\to CF_{3}$	−79.80
7	$CF_{3}+F\to CF_{4}$	−116.80
8	$2CF_{2}\to C_{2}F_{4}$	−77.93
9	$2CF_{3}\to C_{2}F_{6}$	−98.67

^a^*T* = 300 K, at mGGA-M06 L level with ZPE correction and enthalpy correction.

As shown in figure 8, the various free radicals produced by c-C_4_F_8_ reacting stepwise to form CF_4_ and C_2_F_6_ need to absorb 1.47 and 19.6 kcal mol^−1^, respectively, and the generation of C and F requires to absorb 406.79 kcal mol^−1^. From the thermodynamic point of view, there is a dynamic equilibrium process between the various produced radicals, which ensures the insulation performance of the system to a certain extent.

**Figure 9.**
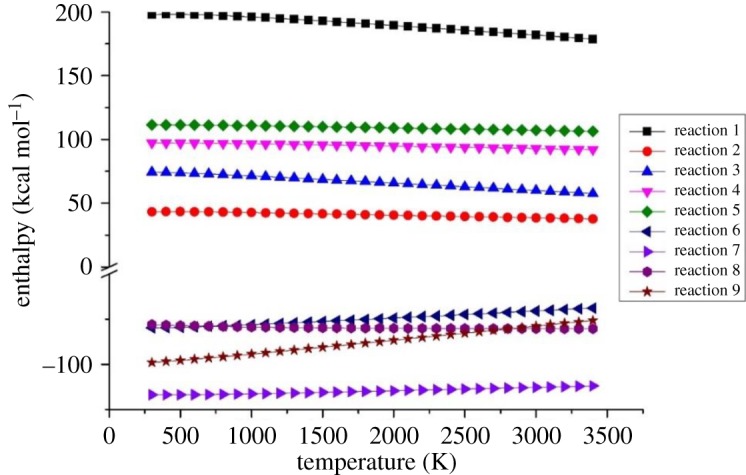
Enthalpy change of proposed reaction paths at 300–3400 K.

In order to further analyse the influence of temperature on the main reaction, the enthalpy of each path at 300–3400 K is also calculated (as shown in figure 9). It can be found that the enthalpy of path 1 and path 3 shows a decrease trend with the increase of temperature, which means the decomposition of c-C_4_F_8_ is more likely to occur under high-temperature conditions. The enthalpy of path 2, 4, 5 and 8 does not change with the increase of temperature, thus the ambient temperature has no obvious effect on these reactions. The reaction enthalpy of path 6 and path 9 decreases with the increasing temperature, indicating that the generation of CF_3_ and C_2_F_6_ occurs with more difficulty at high temperature.

## 4. Conclusion

In this paper, the decomposition process of c-C_4_F_8_ and c-C_4_F_8_/N_2_ gas mixture were explored based on the ReaxFF molecular dynamics method and DFT. It is found that the decomposition of c-C_4_F_8_ mainly produces CF_2_, F, CF_3_, CF, C, CF_4_ and C_2_F_4_. c-C_4_F_8_ can decompose to C_2_F_4_ by absorbing 43.28 kcal mol^−1^, which is the main decomposition path and this process occurs easily under high temperature. There is a dynamic equilibrium process between the various produced radicals, which ensures the insulation performance of system to a certain extent. The decomposition performance of c-C_4_F_8_/N_2_ gas mixture is better than that of pure c-C_4_F_8_ at the same condition, which is suitable to use as a gas-insulated medium in the field of medium voltage (MV) equipment.

## Data accessibility

Our data are deposited at Dryad Digital Repository: http://dx.doi.org/10.5061/dryad.2r984j6 [32].
